# Impact of a hybrid, short-term prehabilitation on patient-reported outcomes in patients undergoing lung resection for non-small cell lung cancer

**DOI:** 10.1093/icvts/ivaf107

**Published:** 2025-04-29

**Authors:** Alice Finch, Jordan Curry, Gowthanan Santhirakumaran, Saif Alshdifat, Jack Jones, Duncan Grant, Riley Cooper, Anthony Assadourian, Adam Januszewski, Sahra Jalali, Kelvin Lau, William Ricketts, Cecilia Pompili

**Affiliations:** Department of Physical Medicine, Rehabilitation and Sports Medicine, St Bartholomew’s Hospital, London, UK; Wolfson Palliative Care Research Centre, Hull York Medical School, University of Hull, Hull, UK; Department of Thoracic Surgery, St Bartholomew’s Hospital, London, UK; Department of Thoracic Surgery, St Bartholomew’s Hospital, London, UK; Department of Physical Medicine, Rehabilitation and Sports Medicine, St Bartholomew’s Hospital, London, UK; Department of Physical Medicine, Rehabilitation and Sports Medicine, St Bartholomew’s Hospital, London, UK; Department of Physical Medicine, Rehabilitation and Sports Medicine, St Bartholomew’s Hospital, London, UK; Department of Physical Medicine, Rehabilitation and Sports Medicine, St Bartholomew’s Hospital, London, UK; Department of Medical Oncology, St Bartholomew’s Hospital, London, UK; Department of Physical Medicine, Rehabilitation and Sports Medicine, St Bartholomew’s Hospital, London, UK; Department of Thoracic Surgery, St Bartholomew’s Hospital, London, UK; Department of Respiratory Medicine, St Bartholomew’s Hospital, London, UK; Department of Thoracic Surgery, St Bartholomew’s Hospital, London, UK; Institute for Clinical and Applied Health Research, Thoracic Surgery, University of Hull, Hull, UK

**Keywords:** prehabilitation, lung cancer surgery, NSCLC resection, patient-reported outcomes (PROs), quality of life, fitness for surgery

## Abstract

**OBJECTIVES:**

We examined the impact of short-term, multimodal prehabilitation on perioperative functional and patient-reported outcomes (PROs) in patients undergoing surgical resection for non-small cell lung cancer (NSCLC).

**METHODS:**

This is a retrospective study with paired comparisons on consecutive patients worked up for surgical resection for suspected NSCLC referred for prehabilitation including exercise, nutritional, and PROs assessment in a single centre from October 2022 to August 2023. Patients participated in a hybrid programme, with twice-weekly, one-to-one sessions combing high-intensity interval-style and strength training with accompanying app-based exercise and lifestyle support. Functional outcomes were assessed via the 6-minute walk test (6MWT) and 1-minute sit-to-stand (1M-STS), and PROs were evaluated using the EuroQol 5-Dimension 5-Level (EQ-5D-5L) and Patient-Generated Subjective Global Assessment (PG-SGA). A multivariable logistic regression analysis identified factors linked to significant PRO improvement.

**RESULTS:**

During the study period, 85 patients were referred, with 98% consenting and 91% (75/85) completing a median of five sessions over 2.5 weeks, with 69% ultimately undergoing surgical resection. There was significant improvement in 6MWT distance (62.8 m, *P* < 0.001), 1M-STS (8.9 repetitions < 0.001), EQ-5D-5L (+6 points, *P* = 0.012) and PG-SGA nutritional status (−0.64 points, *P *= 0.044). Female sex, lower deprivation index (most deprived) and fewer sessions were associated with greater PRO improvements.

**CONCLUSIONS:**

Short-term hybrid prehabilitation for resectable NSCLC improves patient functional and subjective outcomes, particularly among females and those from more deprived areas. This approach appears to enhance preoperative fitness and PROs for patients undergoing surgery potentially reducing postoperative complications and improving postoperative quality of life.

## GRAPHICAL ABSTRACT

**Figure:**
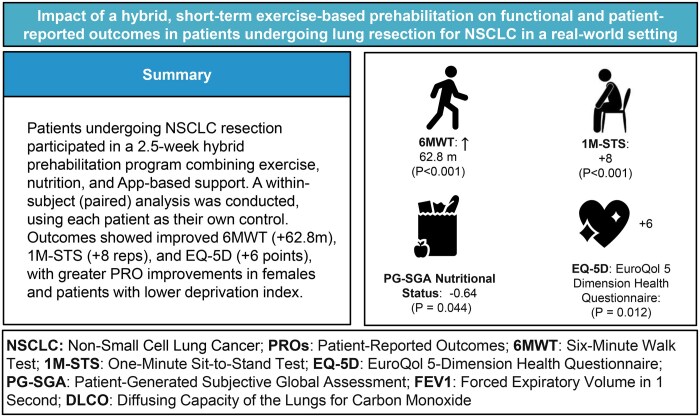

## INTRODUCTION

Surgical resection for early-stage non-small cell lung cancer (NSCLC) remains the gold standard in treatment [1, 2]. However, the physiological demands of lung resection, coupled with frequently coexistent comorbidities of chronic obstructive pulmonary disease (COPD) and cardiovascular disease, present substantial challenges to postoperative recovery. Prehabilitation, a structured programme to optimize a patient’s physical and psychological health prior to surgery, has emerged as a promising strategy to improve functional outcomes and surgical resilience [3].

As part of prehabilitation, the benefits of exercise include enhanced cardiovascular fitness, increased muscle strength and improved endurance, all of which are associated with better surgical outcomes and improved recovery [4]. Furthermore, prehabilitation for those with NSCLC may also improve postoperative health-related quality of life [5].

The fitness assessment process before lung cancer resection relies on standardized assessments, including lung function tests such as forced expiratory volume in 1 second (FEV1) and diffusion capacity of the lungs for carbon monoxide (DLCO) [6]. Cardiopulmonary exercise testing (CPET) is the gold standard for predicting postoperative outcomes [7, 8], but access is limited by capacity constraints. To overcome this issue, simpler field tests such as the 6-minute walk test (6MWT), incremental shuttle-walk test (ISWT) and various sit-to-stand tests can be used as surrogate assessments of functional status prior to and to assess response to prehabilitation. The 1-minute sit-to-stand (1M-STS) test is a tool that evaluates lower limb strength, endurance, and overall functional capacity [9].

Exercise training in the lung cancer preoperative setting has been shown to improve preoperative functional status and, in turn, reduce postoperative pulmonary complications and length of stay [10]. Among various exercise modalities, high-intensity interval training (HIIT) has gained recent attention due to its ability to produce rapid physiological responses in a shorter timeframe [11]. Compared to moderate-intensity continuous training, HIIT has demonstrated greater improvements in peak oxygen uptake (VO_2_peak) [12]. Despite its advantages, HIIT remains underutilized in standard prehabilitation programmes.

In this proof-of-concept study, we aim to investigate the impact of a short-term exercise-based prehabilitation programme on preoperative functional status and patient-reported outcomes (PROs) in patients undergoing lung resection for NSCLC.

## METHODS

A retrospective analysis was conducted of all patients referred for and, ultimately undergoing, prehabilitation for proposed lung cancer surgery at a thoracic surgical centre in London. Patients with planned surgical resection were referred and initially assessed on their exercise and nutritional status along with PROs assessment.

### Referral pathway

The prehabilitation pathway consisted of an initial referral from either a respiratory physician or a thoracic surgeon during the patient’s lung cancer diagnostic pathway. The criteria for referral were broad, with the main emphasis on patients who were likely to be operable but in the relatively high-risk category due to borderline lung function test results and/or reduced functional status according to the ERS/ESTS guidelines [13]. While it is likely that less physiologically limited patients would also benefit from prehabilitation, due to capacity constraints, we primarily aimed for those likely to benefit the most. The patients were approached by the prehabilitation team and were booked for an initial prehabilitation assessment within 3 days of the referral.

### Ethical statement

The initial proposal to implement new prehabilitation services across the local area was completed by a local Cancer Alliance and submitted to NHS England. Once the proposal was accepted and funding received by the Cancer Alliance, funding was provided to three separate NHS Trusts. As the proposal for service development and implementation was completed by the Cancer Alliance to NHS England, ethical approval was not sought and individual patient’s consent for this retrospective analysis was waived.

### Prehabilitation programme

The prehabilitation team consisted of a physiotherapist and four clinical exercise physiologists. The location of the prehabilitation programme was set up to be spread across five different hospital sites to optimize ease of access for patients and adherence. The referred patients underwent an initial trimodal assessment, which included functional, nutritional and psychological assessments. Exercise tolerance and functional capacity were assessed using a 6MWT and 1M-STS, which were chosen pragmatically due to their repeatability and limitations in CPET capacity.

Nutritional status was assessed using the Abbreviated Patient-Generated Subjective Global Assessment (abPG-SGA), which measures oral intake, weight loss, appetite and functional status and is validated for oncology patients [14]. If a patient scored nine or more, indicating they were either moderate or severely malnourished, they were prescribed oral-nutritional supplements, with weight monitored at each appointment (Supplementary Material S1). Nutritional status was reviewed at preoperative assessment to determine any improvement preoperatively, with reduction in abPG-SGA score defining improvement.

Psychological status and quality of life were assessed using the EuroQol 5-Dimension 5-Level (EQ-5D-5L), which has shown to be a simple and easy to administer tool to assess quality of life (QoL), including for those with lung cancer [15], and the Patient Health Questionnaire-4 (PHQ-4) to assess psychological status [16]. The EQ-5D-5 l consists of five dimensions (mobility, self-care, usual activity, pain or discomfort and anxiety or depression), on five levels of severity and a visual analogue scale of 0 to 100 rating of health. We have used the health scale-score in the analysis to better understand the impact of the intervention on the overall QoL.

Patients scoring six or above on the PHQ-4, indicating moderate to severe psychological distress, were referred to the psycho-oncology team.

Finally, a smoking history was taken, and referral to the smoking cessation team was completed for tobacco dependent patients, if consent was gained.

Following the initial assessment, patients were booked for twice weekly, 1-hour one-to-one exercise sessions and a prescribed aerobic-based home exercise programme to complete between sessions. The sessions took place at the hospital gym at the closest site to their home or at a site where another appointment was being held on the same day to reduce travel burden and appointment fatigue and maximize an often-short lead time to surgery. The exercise sessions consisted of combined HIIT, aerobic and resistance training. HIIT has been shown to improve cardiopulmonary fitness [17] with a low risk of adverse events and positive effects on health-related outcomes in a prehabilitation setting [18]. Similarly, resistance and aerobic-style training have been shown to improve muscle mass, fatigue, sleep and quality of life in those with advanced cancer [19]. The exercise intervention was tailored to individual patient’s needs, but followed the following FIIT (frequency, intensity, time, type) principles:

- Frequency: twice weekly, one-to-one sessions with an exercise physiologist plus additional aerobic-based exercises prescribed to be completed at home.
- Intensity: combined HIIT and resistance training sessions in the one-to-one sessions. Clinical reasoning and assessment of past medical history ensured safety of implementing HIIT. In those who may be deemed inappropriate for HIIT, moderate intensity intervals were completed.
- Time: the twice-weekly sessions are 60 min, and additional prescribed home activity aiming to reach the UK chief medical officer’s recommendations for at least 150 min of moderate exercise per week [20].
- Type: whole-body resistance training was implemented. Gym equipment and free weights were used to facilitate multi-joint exercises while applying progressive overload principles. Intensity was monitored using heart rate monitors and rating of perceived exertion (RPE) which facilitated progression of total cardiovascular workload to maximize adaptation.

To maximize adherence, patient-centred goals were set and monitored to support motivation and monitor progress.

Due to short notice changes to scheduling, it was not always possible to plan when a patient’s final preoperative session would take place, but where possible all initial outcome measures were repeated (6MWT, 1M-STS, EQ-5D-5 l, abPG-SGA, PHQ4), and postoperative guidance as per Enhanced Recovery After Surgery (ERAS) guidelines were discussed, including deep breathing exercises, supported cough and importance of early mobilization. Any high-risk patients were discussed with the inpatient physiotherapy team for follow-up postoperatively.

### Statistical analysis

A descriptive statistic was performed to report patient flow and reasons for not undergoing surgery. The normal distribution of numeric variables was assessed using the Shapiro–Wilk test.

Numeric variables were compared by means of paired t-test or Mann–Whitney test in case of non-normal distribution. Categorical variables were tested by means of the Chi-squared test or Fisher’s exact test (where the number of observations was less than 10 in at least one cell). A paired t-test was performed comparing the pre- and postintervention (prehabilitation) 6MWT distance, 1M-STS repetitions and abPG-SGA and EQ-5D-5 l scores (all normally distributed, no missing variables).

A multivariable logistic regression analysis was performed to identify factors associated with a large improvement in EQ-5D-5 l (defined as a difference greater than the value corresponding to the 75th centile of the difference between baseline and post-rehabilitation scores).

The following variables selected for their clinical meaningfulness were initially entered in a stepwise regression, with backward elimination (*P* value for retention 0.1): age, gender, FEV1 and DLCO (both expressed as percentage predicted), body mass index, surgical access, deprivation index and number of prehabilitation sessions. No variables in the model were correlated with a correlation coefficient >0.5 and this ruled out multicollinearity.

All tests were performed using Stata 15.0 statistical software (Stata Corp, College Station, TX, USA).

## RESULTS

We identified a total of 85 patients who were provisionally planned for lung resection and were referred to the prehabilitation team from October 2022 to August 2023. Patients underwent a median of five session (interquartile range [IQR] 4–8.5) one-to-one exercise sessions over 21 days.

Of the 85 patients referred, all were approached by the prehab team and 98% consented to prehabilitation. Two patients declined the service, one due to the psychological distress of a new cancer diagnosis and the other not wanting to travel for additional appointments.

**Figure 1:**
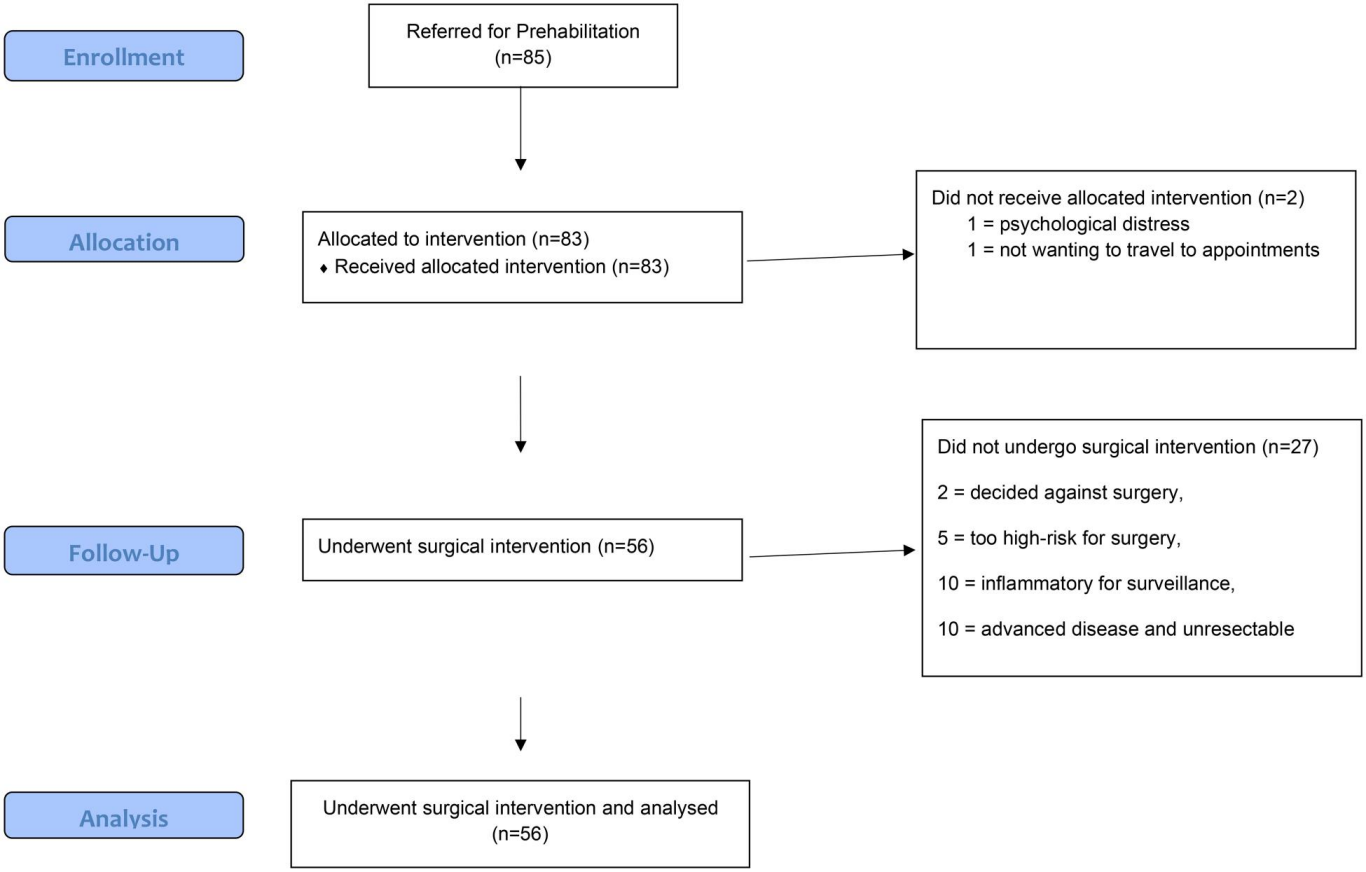
CONSORT flow diagram

All 83 enrolled patients completed at least two sessions, with initial and preoperative assessments completed. Of those patients, 69% (56) underwent surgical resection (see Fig. 1 for CONSORT flowchart). Reasons for not undergoing surgery were identified as follows: two patients decided against surgery entirely, five patients were deemed too high-risk to operate following a high-risk multi-disciplinary team (MDT) clinic (all undergoing systemic- or radio-/ablative-therapy instead), and, following further work-up and staging investigations, 10 patients were felt to have likely benign disease and further 10 were subsequently upstaged or diagnosed with mesothelioma, making them not suitable for resection.

Lung resections were performed via minimally invasive thoracoscopic surgery including video-assisted thoracoscopy surgery (VATS: 48.2%) and robotic-assisted thoracoscopy surgery (RATS: 44.6%) in 93% (52/56) and open thoracotomy in 7% (4/56) of patients. Lobectomy was performed in 23 patients (41%), segmentectomy in 15 (27%) and 18 patients underwent wedge resection (Table 1).

**Table 1:** Patient demographics and clinical characteristics

Patient demographic	Prehab group (*n* = 56)
Age (years)	68.4 (SD 9.1)
Sex	Female 29
Deprivation index (median)	3 (IQR 2–8)
Baseline lung function	
FEV1 (% Predicted)	78.7 (SD 25.6)
DLCO (% Predicted)	68.6 (SD 20.5)
Surgery	
Minimally invasive surgery	52 (92.86%)
RATS	27 (48.21%)
VATS	25 (44.64%)
Open thoracotomy	4 (7.14%)
Lobectomy	23 (40.07%)
Sublobar resection	33 (58.93%)
Segmentectomy	15(26.79%)
Wedge resection	18 (32.14%)
Cardio-pulmonary complications at 30 days	3.6%

All variables are normally distributed except for the deprivation index which is therefore expressed as median and interquartile range. IQR: interquartile range; SD: standard deviation; FEV1: forced expiratory volume in 1 second; DLCO: diffusion capacity of the lung for carbon monoxide; RATS: robotic-assisted thoracic surgery; VATS: video-assisted thoracic surgery.

### Patients’ characteristics

The patients’ baseline characteristics are shown in Table 1.

Median age of patients who underwent prehabilitation was 68 years (IQR 62–74). The mean Charlson comorbidity index for those who underwent prehabilitation was 5.7. In the prehabilitation cohort who underwent lung resection, two patients required readmission due to pneumonia, no other serious complications were noted.

A logistic regression analysis was performed to identify factors associated with a large improvement in EQ-5D-5 l score. After adjusting for other patient-related confounders, female sex, lower deprivation index (most socioeconomically deprived patients) and a smaller number of sessions are associated with higher improvement in quality of life (Table 2).

**Table 2:** Results of the logistic regression analysis to identify factors associated with postoperative clinically meaningful changes in quality of life (EQ-5D-5 l score)

Variables	Regression coefficients	OR	SE	*P* value	95% CI
Sex (male)	−1.23	0.289	0.19	0.062	0.078 to 1.064
Deprivation index	−0.37	0.69	0.12	0.034	0.489 to −0.971
Number of sessions	−0.2	0.811	0.08	0.035	0.667 to 0.985

SE: standard error; CI: confidence interval; OR: odds ratio.

Significant improvements were observed in both functional and subjective results after prehabilitation (Table 3). The mean 6MWT increased from 339 m (SD 16 m) before prehabilitation to 401 m (SD 16 m) after, with a mean change of 62.8 m (SD 47.0 m) (*P* < 0.001, Cohen’s d = 0.59), more than the minimally significant distance for 6MWT in lung cancer [19]. The 1M-STS demonstrated significant improvement, increasing from 21.1 (SD 8.8) repetitions to 30.0 (SD 11.7) repetitions, with a mean change of 8.9 (SD 7.1) repetitions (*P* < 0.001, Cohen’s d = 0.87), demonstrating a large effect size (>0.80) (Table 3).

**Table 3:** Summary of before and after prehabilitation results

	Before prehab	After prehab	Mean change	*P* value	Effect size (Cohen’s d)
6MWT distance (m)	339 (16)	401 (16)	62.8 (47.0)	<0.001	0.59
1M-STS	21.1 (8.8)	30.0 (11.7)	8.9 (7.1)	<0.001	0.87
abPG-SGA	2.56 (3.7)	1.92 (3.5)	−0.64 (2.2)	0.044	−0.25
EQ-5D-5 l score	62.6 (14.9)	68.6 (13.3)	6.0 (16.3)	0.012	0.45

Results are expressed as means and standard deviations. As reference, for effect sizes 0.2 is considered small, 0.5 medium and 0.8 large. All variables are normally distributed. 6MWT: 6-minute walk test; 1M-STS: 1-minute sit-to-stand test; abPG-SGA: abbreviated patient-generated subjective global assessment; EQ-5D-5L: EuroQol 5-Dimension 5-Level score; Prehab: prehabilitation.

Nutritional status, assessed using the abPG-SGA, demonstrated borderline significant improvement, with scores decreasing from 2.56 (SD 3.7) to 1.92 (SD 3.5) (mean change: −0.64 [SD 2.2], *P* = 0.044, Cohen’s d = −0.25). Health-related quality of life, measured by the EQ-5D-5 l score, improved from 62.6 (SD 14.9) to 68.6 (SD 13.3), with a mean change of 6.0 (SD 16.3) (*P* = 0.012, Cohen’s d = 0.45), demonstrating a low-moderate effect size (Table 3).

## DISCUSSION

The statistically significant improvements demonstrated in the 6MWT of 62.8 m and 1M-STS of 8.9 repetitions suggest that supervised, combined high-intensity interval and resistance training twice weekly in a real-world setting for as little as 3 weeks is an effective intervention to improve preoperative functional status for patients undergoing lung cancer resection surgery, with an acceptable rate of adverse outcomes. We did not complete further postoperative follow-up; however, Machado *et al.* [21] demonstrated in a multicentre randomized control trial that the benefits of prehabilitation remained at 1 month after surgery, suggesting that our service may infer long-lasting benefits for our patients beyond simply improved perioperative outcomes.

PROs also displayed an improvement in patients’ psycho-physical domains, which is an important component of a trimodal prehabilitation programme. Poor preoperative psychological status has been suggested to impact postoperative recovery, including increased length of stay, incidences of chronic pain and slower return to work [22]. The improvements demonstrated in the psycho-physical domains may indicate that prehabilitation could further support postoperative recovery by reducing psychological stressors.

Abridged PG-SGA malnutrition screening showed only a borderline significant improvement. A request for oral nutritional supplements and referral to community-based dietitian service was requested to the local general practitioner for those screened who scored >9 on the abPG-SGA (six patients). Despite this, no patients were seen by a community-based dietitian service ahead of surgery due to a short lead time for surgery and extensive waiting lists for local services; with this in mind, we have successfully applied for funding for a 2-year pilot of adding a dietician to our service.

We had a high patient adherence rate in the prehabilitation programme, demonstrating the intervention being both feasible and acceptable to the participants. There are several factors that likely impacted this high adherence rate. Having access to five different hospital gyms likely increased adherence, as this meant people could access the service closer to home or in conjunction with other appointments. This reduces travel time and improves ease of access; and qualitative reviews have stated access to services being a facilitator to participation in prehabilitation and difficulty with transport a barrier [23]. The supervised, guided one-to-one sessions may have also increased adherence rates, as having individualized programmes adapted to the individual’s needs and abilities has also been described as an essential facilitator to prehabilitation [24]. The endorsement of prehabilitation by the patient’s, surgeons, physicians, nurses and oncologists will likely have impacted adherence further, as encouragement by healthcare professionals has also been reported as a motivating factor for participation in prehabilitation [23]. While prehabilitation is often targeted at patients with lower baseline fitness, those with higher functional capacity may also derive benefits. Evidence suggests that even well-conditioned individuals can experience postoperative deconditioning, and structured prehabilitation may help mitigate this decline. Additionally, prehabilitation can enhance physiological reserves, optimize nutritional status and provide psychological support, all contributing to improved surgical recovery. Therefore, a real-world approach to prehabilitation should include all patients undergoing major lung resection, ensuring individualized adaptations based on baseline fitness levels.

Looking at future implementation challenges, in light of the results of the Checkmate-816 study [25], there is likely to be an increase in the use of neoadjuvant treatment regimens in patients with NSCLC, potentially increasing need for prehab services, not just optimizing patients prior to treatment, but also maintaining or furthering that improvement during the neoadjuvant treatment period.

The limitations of the study remain the retrospective analysis of the data with limited follow-up. Despite the benefits of prehabilitation across outcome measures compared to baseline, long-term follow-up is required to assess for sustained benefit. Our prehabilitation referral pathway encouraged early referral to maximize the duration of the programme, with high uptake; however, this did mean that some patients later came off the programme either due to having a more advanced disease or benign pathology. The referral pathway also relied on referrals from a surgeon of respiratory physician, with broad referral criteria. Data were not collated as to those who underwent surgery but were not referred into the prehabilitation service. More details on reasons for declining or not further participation to the programme are needed in future evaluation, to better understand compliance and areas of improvement. Feedback from the functional outcome measures repeated by the prehabilitation team may serve as an additional preassessment tool and could be utilized by the wider MDT when assessing risk and ensuring all people who would benefit from prehabilitation are referred. Due to limited availability of the CPET testing, this study has not assessed the patients with the gold standard physiological testing modality, so future implementation should also look at integrating this into the prehabilitation pathway.

In summary, this retrospective single-centre study illustrated the real-world implementation targets for prehabilitation. It demonstrates high adherence with twice-weekly, one-to-one supervised exercise sessions, improving preoperative functional status in just 3 weeks. Further qualitative exploration would be valuable to identify and understand the barriers to participation, providing deeper insights into potential challenges and opportunities for improving engagement.

These findings support the real-world practice of prehabilitation for lung cancer surgery and illustrate the impact of its patient-centred implementation. Future prospective studies will need larger sample sizes and longer-term follow-up to analyse the prehabilitation programme’s effect on postoperative psychological and physiological outcomes.

## Supplementary Material

ivaf107_Supplementary_Data

## Data Availability

The data underlying this article will be shared on reasonable request to the corresponding author.

## ABBREVIATIONS

CPET: Cardiopulmonary exercise testing
EQ-5D-5L: EuroQol 5-Dimension 5-Level
HIIT: High-intensity interval training
NSCLC: Non-small cell lung cancer
PRO: Patient-reported outcome
PG-SGA: Patient-Generated Subjective Global Assessment
6MWT: 6-minute walk test
1M-STS: 1-minute sit-to-stand
