# Supplementary material for: Impact of a hybrid, short-term prehabilitation on patient-reported outcomes in patients undergoing lung resection for non-small cell lung cancer
Source: Interdiscip Cardiovasc Thorac Surg. 2025 Apr 29;40(6):ivaf107. doi: 10.1093/icvts/ivaf107 (PMC12167633; doi:10.1093/icvts/ivaf107)
Supplement: ivaf107_Supplementary_Data [file ivaf107_supplementary_data.docx]

**Appendix**

*Appendix 1: The malnutrition screening pathway and onward referral template to the GP for oral nutritional supplements.*

**Nutritional Screening and Assessment Pathway for Oncology Prehab**

All patients attending the prehab are nutritionally screened. This is delivered as part of their baseline assessment by their exercise physiologist, who have all completed additional training in nutritional screening. This will include:

- BMI (weight and height)
- Abridged Patient Generated-Subjective Global Assessment tool (abPG-SGA tool)

**Action Plans**

| **BMI** | **abPG-SGA Score** | **Literature to be Provided** | **Actions** |
| --- | --- | --- | --- |
|  | 0-3 | Macmillian Cancer Support –“Healthy Eating and Cancer” |  |
| BMI 18.5-20kg/m2 | 4-8 | Macmillan Cancer Support – “The Building Up Diet” / “Eating Problems and Cancer” | Suggest over the counter supplements i.e. Complan, Meritene |
| BMI >18.5kg/m2 | 9+ | Macmillan Cancer Support – “The Building Up Diet” / “Eating Problems and Cancer” | Per score 9+  Letter to be sent to GP recommending dietitian referral and consideration of first line oral nutritional supplements  ?Contact clinical team |

**Frequency of Screening**

- Initial assessment

**Other Anthropometric Measures**

- MUAC
- Hand grip strength

**Draft GP Letter**

Dear GP

This patient seen by X as part of their prehabilitation assessment prior to planned oncology treatment.

As part of this assessment a nutritional screen has been carried out.

This patient has been identified as high risk of malnutrition, the details of which are below:

Weight:

BMI:

Weight Loss:

abPG-SGA score (9+ indicates critical need for a dietitian):

Unfortunately, there is no dietetic service within the prehabilitation pathway in North East London and therefore please consider completing the following:

- Referral to your local community dietetic service
- Prescription of first line oral nutritional supplements twice per day for 1 month only:
  - Powder based supplement: Aymes Shake (57g sachet) (1 box contains 7 sachets)
  - Ready to drink: Aymes Complete (200ml bottle)

This patients clinical team have also been made aware of the above nutrition screening score.

Please do not hesitate to contact us should you have any queries.

Yours sincerely, X
